# 

**Published:** 1890-04

**Authors:** 


### CONTENTS.

ORIGINAL ARTICLES—
Report of the Medical Director of the Savannah Sanitary Association —By J. C. LeHardy, M. D., Med. Direc. S. S. A., Savannah, Ga.... 149
Relaxation of the Utero-Sacral Ligaments—An Outline Intended Only to Introduce the Subject for Discussion in Atlanta Society of Medicine March 4th, 1890—By Geo. H. Noble, M. D., Atlanta, Ga....... 154
The Marvelous in Medicine—By E. VanGoidtsnoven, A. M., M. D., Atlanta, Ga.........

CLINICAL DEPARTMENT—
Removal of a Large Sarcoma Parotid Gland-Ligature of the External Carotid Artery an l Lateral Ligature of the Internal Jugular Vein—By John A. Wyeth, M. D., New York, N. Y................ 160
A Case of Almost Total Destruction of the Iris; Recovery with Perfect Vision—By R. O. Cotter, M. D., Macon, Ga..................... 161

CORRESPONDENCE—Our New York Letter—Vienna Letter...........163 166

EDITORIAL—
Higher Education —Southern Medical College Commencement.—Atlanta Medical College Commencement.—Concerning the Tenth International Medical Congress.—Invitation for an International Medical and Scientific Exhibition.—Program of the Meeting of Medical Association of Georgia, at Brunswick, Ga., April 16th, 17th and 18th, 1890.........170 178

PERSONAL NOTES........ 179

PRACTICAL NOTES........ 180

THE ATLANTA SOCIETY OF MEDICINE........ 182

BOOK REVIEWS........ 184

SELECTIONS AND ABSTRACTS—
Temporary Resection of the Cranial Bones as a Substitute for Trephining.—On the Continuous Use of Blue Mass in Small Doses.—On Some Practical Points in Treatment of Syphilis.—Pneumonia in Children.—Death by Chloroform.—Dry Operations.—On the Treatment of Peritonitis.—Practical Points About Surgical Dressings.—Operation for Undescended Testicle and Hernia.—A Pin in the Right Rronchus.—Treatment of Tonsilitis........186 196
Method of Rendering Sponges Aseptic........ 166
The Period for Surgical Interference in Acute Intestinal Obstruction —To Remove Powder Stains........ 183

SPECIAL NOTES...........197 198

